# Llaima volcano dataset: In-depth comparison of deep artificial neural network architectures on seismic events classification

**DOI:** 10.1016/j.dib.2020.105627

**Published:** 2020-04-30

**Authors:** João Paulo Canário, Rodrigo Fernandes de Mello, Millaray Curilem, Fernando Huenupan, Ricardo Araujo Rios

**Affiliations:** a Department of Computer Science, Federal University of Bahia, Brazil; b Institute of Mathematics and Computer Science, University of São Paulo, Brazil; c Department of Electrical Engineering, Universidad de La Frontera, Chile

**Keywords:** Volcano Monitoring, Time Series Analysis, Time Series modelling, Neural Network

## Abstract

This data manuscript presents a set of signals collected from the Llaima volcano located at the western edge of the Andes in Araucania Region, Chile. The signals were recorded from the LAV station between 2010 and 2016. After individually processing and analyzing every signal, specialists from the Observatorio Vulcanológico de los Andes Sur (OVDAS) classified them into four class according to their event source: i) Volcano-Tectonic (VT); ii) Long Period (LP); iii) Tremor (TR), and iv) Tectonic (TC). The dataset is composed of 3592 signals separated by class and filtered to select the segment that contains the most representative part of the seismic event. This dataset is important to support researchers interested in studying seismic signals from active volcanoes and developing new methods to model time-dependent data. In this sense, we have published the manuscript “In-Depth Comparison of Deep Artificial Neural Network Architectures on Seismic Events Classification” [1] analyzing such signals with different Deep Neural Networks (DNN). The main contribution of such manuscript is a new DNN architecture called SeismicNet, which provided classification results among the best in the literature without demanding explicit signal pre-processing steps. Therefore, the reader is referred to such manuscript for the interpretation of the data.

Specifications table

**Table:** 

Subject	*Environmental Science and Artificial Intelligence*
Specific subject area	*Application of neural networks in the modelling of seismic patterns*
Type of data	*1D Time-Frequency Signal*
How data were acquired	*The signals produced by different seismic events were collected from the Llaima volcano located at the western edge of Andes in Araucania Region (S 38o41′ - W 71o44′), Chile. The state agency responsible for monitoring this volcano and processing its signals is the Observatorio Vulcanológico de los Andes Sur (OVDAS).*
Data format	*Raw signals*
Parameters for data collection	*The signals were collected from LAV, that is one of the seven seismic stations of Llaima, being recorded in terms of the Z-vertical component from 2010 to 2016, sampled at 100Hz and filtered using a 10th-order Butterworth bandpass filter in range* [1, 10] *Hz in order to preserve the bandwidth that contains the range of interest. The Z-vertical component contains all the necessary information to classify the event types, according to the specialists from OVDAS.*
Description of data collection	*The dataset was created from signals collected by monitoring stations located at the Llaima volcano (Chile). In summary, the signals represent activities related to the following events:* • *Volcano-Tectonic (VT): events associated with brittle failure of rocks inside of the volcanic building, which is the same type of event that happens along purely tectonic faults (e.g. the San Andreas Fault);* • *Long Period (LP): events usually observed before volcanic eruptions, being an important indicator of relevant imminent activities;* • *Tremor (TR): events that produce continuous and high-amplitude signals originated from several different processes, such as long-lived resonance due to extended flow of magma movement through cracks;* • *Tectonic (TC): events are not related to volcanic activities, being a typical result of the dynamics of geological faults.*
Data source location	*Llaima Volcano located at the western edge of Andes in Araucania Region (S 38^o^41′ - W 71^o^44′), Chile*
Data accessibility	*The data is available at http://dx.doi.org/10.17632/dv8nwdd36k.1*
Related research article	Canário, J. P., de Mello, R. F., Curilem, M., Huenupan, F., Rios, R. A. (2020). In-Depth Comparison of Deep Artificial Neural Network Architectures on Seismic Events Classification. *Journal of Volcanology and Geothermal Research.*

## Value of the data

• The data can inspire new methods to detect and classify signals.
• Our data is helpful to understand different patterns from volcano seismic events.
• The data is important to compare the behavior of similar events from different active volcanoes.

## 1 Data description

**Fig. 1:**
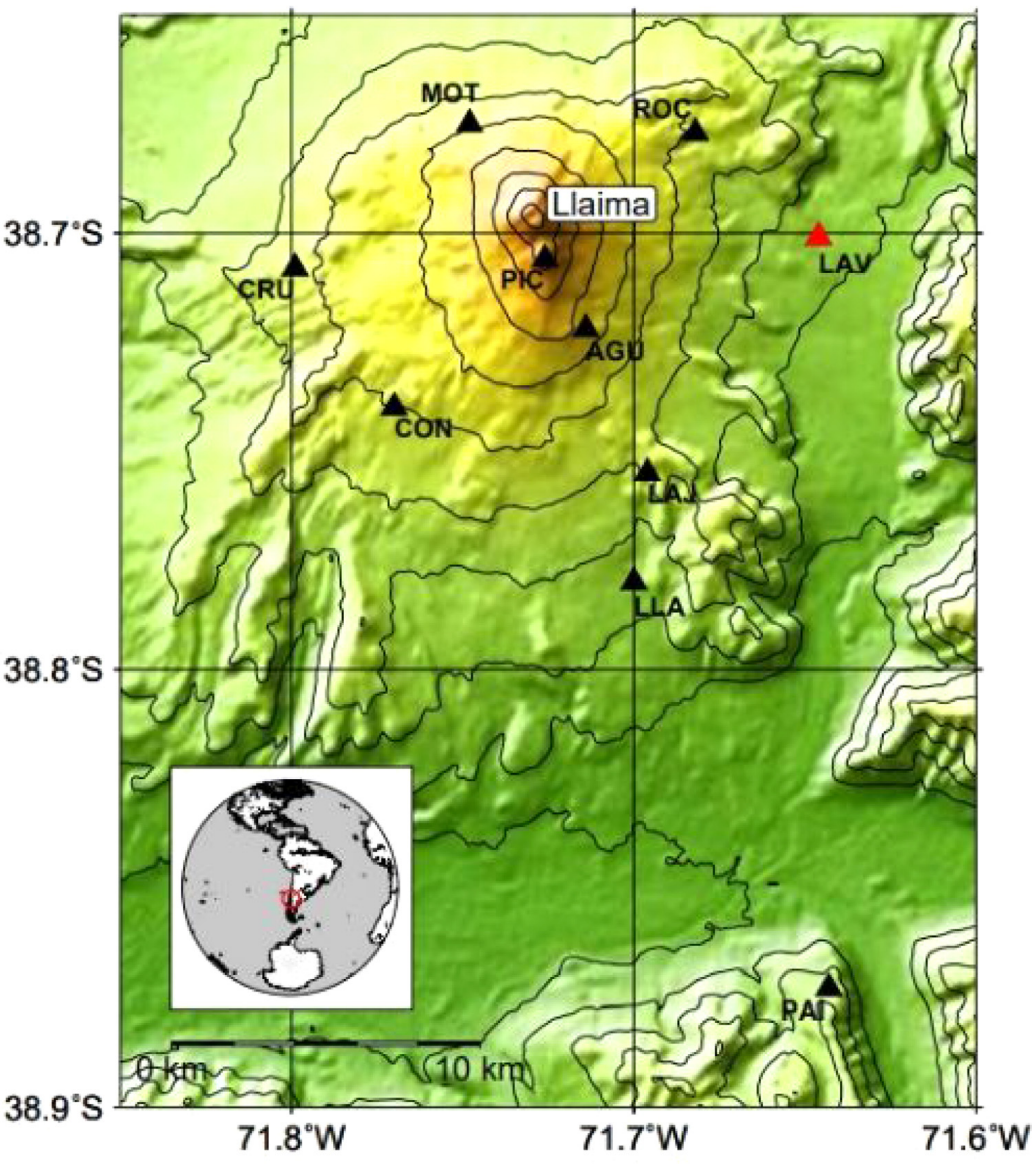
Llaima volcano and its seismic stations [3].

The signals were collected from the Llaima volcano located at the western edge of Andes in Araucania Region (S 38^o^41′ - W 71^o^44′), Chile. Due to its location, Llaima is considered a touristic attraction surrounded by villages, whose productive activity is mainly farming and livestock. Aiming at providing some security level for the people living in the neighborhood, there is a state agency, called OVDAS (Observatorio Vulcanológico de los Andes Sur), that monitors not only Llaima but also other 42 volcanoes over the whole country. In particular for Llaima, OVDAS performs constant surveillance with 9 stations that continuously gather seismic activity with a 24/7 monitoring service as represented in Fig. 1.

In particular, the data presented here takes into account the signals under the types Volcano-Tectonic (VT), Long Period (LP), Tremor (TR) and Tectonic (TC) collected from LAV, one of the seven seismic stations of Llaima, given it was the most complete dataset studied by the specialists from OVDAS, being recorded in terms of the Z-vertical component from 2010 to 2016, sampled at 100Hz and filtered using a 10th-order Butterworth bandpass filter in range [1, 10] Hz in order to preserve the bandwidth that contains the range of interest. The Z-vertical component contains all the necessary information to classify the event types, according to the specialists from OVDAS [1,3]. As a preprocessing step, a total of 3592 signals were normalized using their maximal values and organized into four classes with the following amounts: VT - 304, LP - 1310, TR - 490, and TC - 1488.

**Fig. 2:**
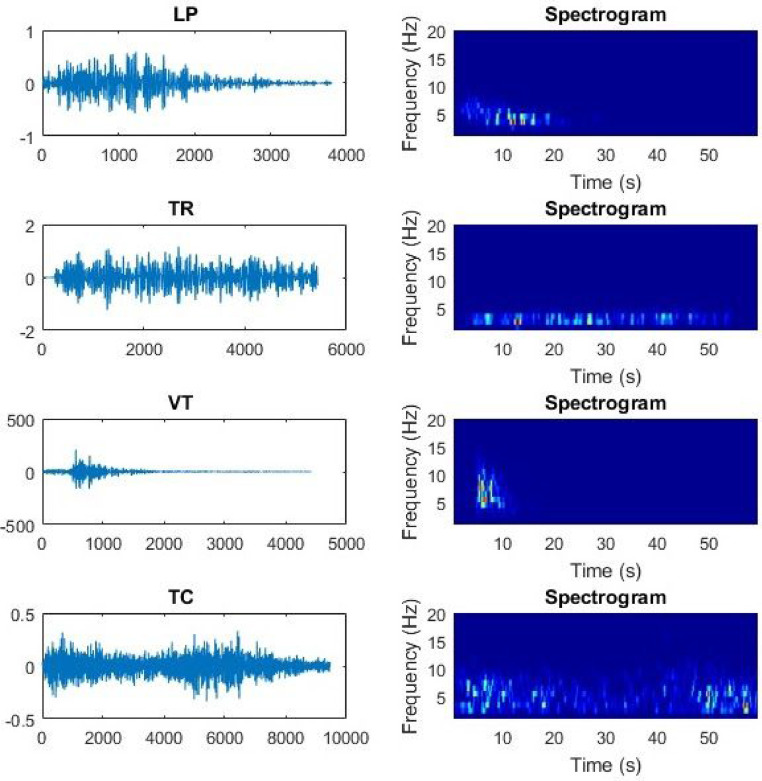
Samples of Llaima signals to represent every seismic event class: (A) LP; (B) TR; (C) VT; and (D) TC.

As mentioned in the Table of Specifications and discussed in [1,3], Volcano-Tectonic (VT) refers to brittle failure of rocks inside of the volcanic building, which is the same type of event that happens along purely tectonic faults. VT events are also resultant of normal tectonic forces inside volcanoes due to the stress caused by the movement of fluids into pre-existing cracks. This sort of activity presents a frequency pattern with a broadband spectrum that may reach 10 Hz. Events under the type Long Period (LP) correspond to the resonating of magma and gases inside volcanic conduits toward the surface, whose spectral pattern is narrower than VT and it is mainly bounded in [0.5, 5] Hz. LP events are usually observed before volcanic eruptions, being an important indicator of relevant imminent activities. However, its occurrence is also part of the normal background seismicity at many volcanoes. Non-magmatic processes may also produce LP events, such as the case of glacier movements. Tremor (TR) are continuous and high-amplitude signals produced by several different processes, such as long-lived resonance due to extended flow of magma movement through cracks, the continuous incidence of other events such as VT and LP when closely spaced over time. Their broadband spectrum is usually in range [0.5, 3.0] Hz, being slowly attenuated at the end of the event. At last, Tectonic (TC) events are not related to volcanic activities, being a typical result of the dynamics of geological faults. TCs may be the result of local, regional or even distant activities in terms of the epicenter location. When the TC event is detected far from the epicenter, it has lower frequencies than nearby ones. According to the proximity of the source, TC could be misclassified as LP or VT. The spectral content of TC is similar to VT, being characterized by an impulsive beginning and an exponential decay, on the other hand it typically carries more energy which is made evident by analyzing the signal amplitudes. Fig. 2 illustrates an example of evey seismic class available in our dataset along with their spectrogram to better describe the signal behavior.

## 2 Experimental design, materials, and methods

As discussed in [1,2], these volcano signals were analyzed using different approaches: raw data, spectrograms, and Wavelets. Experiments presented in [2] were designed to analyze the DNN performance while discriminating seismic activities through two common network architectures: a 2D Convolutional Neural Networks (CNN) and the Long Short-Term Memory (LSTM) network [2]. In manuscript [1], we extended the analysis presented in [2] by comparing those two architectures against the Multilayer Perceptron (MLP), which would be the simplest and most common baseline to take into account. Finally, a new architecture was proposed in [1], referred to as SeismicNet, allowed to accurately classify signals without demanding explicit signal pre-processing steps though.

As commonly performed in the literature of artificial neural networks, we analyzed in [1] multiple architecture settings in terms of their loss (or accuracy performance if we consider its complement) in order to estimate the best as possible configurations to address the problem of interest. The obtained results were evaluated by using measures traditionally computed to assess supervised learning tasks. Firstly, the generalization capability of our predictive models was studied by sampling the original dataset using a 10-fold cross-validation strategy. Then, the results were organized into one-vs-all contingency matrices containing the number of true positive (TP), true negative (TN), false positive (FP), and false negative (FN). Based on such matrices, we calculated four measures: i) Accuracy; ii) Error; iii) Specificity; and iv) F1-score. In addition to these indices, the Kappa coefficient was also used to measure the general agreement between our classification system and experts, emphasizing the results were not obtained by chance.

According to the results presented in [1], one may notice the adjusted models were capable of modeling and learning from seismic signals, by providing high accuracies within the interval of 96.2% and 98.10%. Moreover, the other classification measures (Recall, Specificity, Kappa, and F1-score) were greater than 90%, whereas the error for all models were, at most, 3.1%. It is also important to emphasize the outstanding results obtained with SeismicNet, showing classification rates greater than 90%, without transforming the signals to images (outputs from Fourier and Wavelet transforms) before proceeding with the training phase.
